# Functional Evaluation, Antioxidant, Antimicrobial, Antibiofilm, and Haemolytic Capacity of *Calathea lutea* (Bijao) and *Calathea inocephala* (Shutupipanga) Leaves

**DOI:** 10.3390/antiox15030274

**Published:** 2026-02-24

**Authors:** Elena Coyago-Cruz, Arianna Mayorga-Ramos, Gabriela Méndez, Lizbeth Alpusig-Guanoluisa, Felipe Rivera-Rueda, Johana Zúñiga-Miranda, Carlos Barba-Ostria, Jorge Heredia-Moya

**Affiliations:** 1 Carrera de Ingeniería en Biotecnología, Universidad Politécnica Salesiana, Sede Quito, Campus El Girón, Av. 12 de Octubre N2422 y Wilson, Quito 170143, Ecuador; 2 Centro de Investigación Biomédica (CENBIO), Facultad de Ciencias de la Salud Eugenio Espejo, Universidad UTE, Quito 170527, Ecuador; 3 Escuela de Medicina, Colegio de Ciencias de la Salud Quito, Universidad San Francisco de Quito (USFQ), Quito 170901, Ecuador; 4 Instituto de Microbiología, Universidad San Francisco de Quito (USFQ), Quito 170901, Ecuador

**Keywords:** functional foods, natural antimicrobials, biocompounds, antioxidant activity, anthocyanins, SD3

## Abstract

Amazonian communities traditionally use plant leaves to wrap food; however, there is little information available on the species and their health benefits. This study aimed to characterise the physicochemical properties of the samples, including pH, total soluble solids, total titratable acidity, moisture content, ash, and mineral composition determined by atomic absorption spectroscopy. Major bioactive compounds, including vitamin C, organic acids, carotenoids, chlorophylls and derivatives, and phenolic compounds, were determined by liquid chromatography. The antioxidant potential was examined using ABTS and DPPH, antimicrobials (bacteria and fungi), biofilm inhibition (bacteria), and the haemolytic activity of *Calathea lutea* and *Calathea inocephala* leaves was evaluated. *C. lutea* showed high iron (2930.0 mg/100 g DW), vitamin C (4.6 mg/100 g DW), and tartaric acid (722.3 mg/100 g DW). *C. inocephala* showed high lutein (83.5 mg/100 g DW) and pheophytin b (177.5 mg/100 g DW). Major phenolics included caffeic acid (16,996.3 mg/100 g DW). Extracts at 1 mg/mL inhibited multidrug resistance in *Enterococcus faecalis* and *Enterococcus faecium* and showed strong antibiofilm activity against *Listeria monocytogenes*. The antioxidant activity was 4.6 mmol TE/100 g DW in the DPPH method, and the compound was haemocompatible at concentrations below 600 µg/mL. These findings highlight its biotechnological potential and importance for sustainable community use.

## 1. Introduction

The Amazon rainforest is home to an extraordinary biodiversity of plant and animal species. It offers a vast reserve of natural resources with promising applications in the food and pharmaceutical industries. Plants are a source of bioactive compounds and have long been recognised for their pharmacological potential, including antioxidant, antimicrobial, and therapeutic properties, often with minimal side effects [1]. Despite their ethnobotanical relevance, many Amazonian species remain underexplored in terms of their chemical composition and functional properties, which makes them unknown [2].

In this context, two species of Marantaceae in the genus *Calathea*, native to South America, are of particular interest. These species have been little studied and are mainly used for food coating, providing characteristic flavours, namely *Calathea lutea* (Aubl.). Schult., commonly known as shutupipanga, and *Calathea inocephala* (Kuntze) H. Kenn. & Nicolson, known locally as bijao. Both species are acaulescent herbs reaching up to 3 m in height, characterised by broad, elliptic leaves, traditionally used in culinary practices to pack food items and in ethnomedicine, particularly for mental health disorders [3,4]. *Calathea lutea* is native to tropical regions of the Americas, including Ecuador, and *C. inocephala* shares similar ecological and morphological characteristics. It is considered a non-timber species in tropical forests, and in other areas, it is used as an outdoor and indoor plant [3,4].

Despite the widespread traditional use of these leaves in Amazonian communities, scientific data on the nutritional composition, bioactivity, and industrial potential of these plants remain scarce. This gap is particularly concerning in the context of rising antimicrobial resistance, exacerbated by the indiscriminate use of antibiotics in humans and veterinary medicine, an issue intensified during the COVID-19 pandemic [5]. Thus, in some regions, the lack of stringent regulatory frameworks has further accelerated the spread of resistant microorganisms, highlighting the urgent need for alternative therapeutic agents derived from natural sources [6]. Extracts and components derived from species of the Marantaceae family have shown effectiveness against a range of microorganisms, suggesting their potential as sources for the development of natural antimicrobial agents [7].

Among antimicrobial strategies, a promising alternative is to inhibit the formation of microbial biofilms, which are key factors in the persistence of infections and the development of antibiotic resistance. Natural plant extracts have shown potential as anti-biofilm agents, capable of disrupting biofilm architecture or preventing its establishment [8]. However, the biofilm-inhibitory capacity of *C. lutea* and *C. inocephala* has not yet been characterised. To address this issue, the present study evaluates the physicochemical characteristics, bioactive compounds, and antioxidant, antimicrobial, biofilm-inhibiting, and haemolytic activities of the leaves of *C. lutea* and *C. inocephala*. This study generates preliminary evidence that may guide further research into the functional and nutraceutical implications of these bioactive compounds in human health.

## 2. Materials and Methods

### 2.1. Reference Standards

The high-purity analytical standards used in this research were purchased from Sigma–Aldrich (Merck, Darmstadt, Germany). These included vitamin C (99.8%, *L*-(+)-ascorbic acid); organic acids (100.8% citric acid, 99.0% malic acid, and 99.5% *L*-(+)-tartaric acid); carotenoids (97.0% astaxanthin, 100.0% lutein, 90.0% violaxanthin, 100.0% zeaxanthin, 96.0% trans-β-apo-8-carotenal, 95.0% α-carotene, 93.0% β-carotene, 97.0% β-cryptoxanthin, and 98.0% lycopene); chlorophylls and derivatives (100.0% chlorophyll a, 90.0% chlorophyll b, and 90.0% pheophytin a); and phenolic compounds (98.0% caffeic acid, 95.0% chlorogenic acid, 97.0% chrysin, 100.0% ferulic acid, 100.0% gallic acid, 98.0% 2,5-dihydroxybenzoic acid, 99.0% 3-hydroxybenzoic acid, 97.0% kaempferol, 98.0% luteolin, 99.0% *m*-coumaric acid, 95.0% naringin, 97.0% *o*-coumaric acid, 98.0% *p*-coumaric acid, 99.0% *p*-hydroxybenzoic acid, 95.0% quercetin, 94.0% rutin, 99.0% shikimic acid, 95.0% syringic acid, and 97.0% vanillic acid). Trolox (98.0%) was also used to evaluate antioxidant activity. Mineral standards (calcium, iron, magnesium, potassium, and sodium) at a concentration of 100 µg/mL were purchased from AccuStandard (AccuStandard, Inc., New Haven, CT, USA).

On the other hand, the reference microbial strains *Candida albicans* ATCC 1031, *Candida tropicalis* ATCC 13803, *Escherichia coli* ATCC 8739, *Pseudomonas aeruginosa* ATCC 9027, *Staphylococcus aureus* ATCC 6538P, and *Streptococcus mutans* ATCC 25175 were obtained from the American Type Culture Collection (ATCC, Manassas, VA, USA).

### 2.2. Plant Material and Physicochemical Analysis

**Figure 1:**
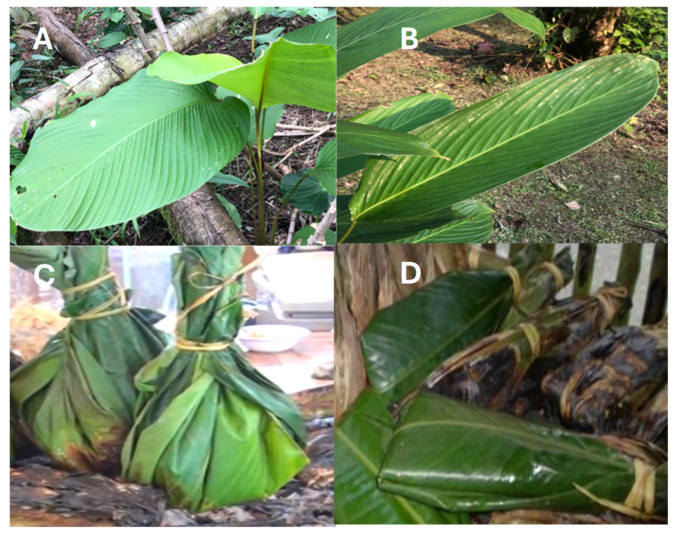
Photograph of leaves of bijao and shutupipanga and their uses in Amazonian cuisine. Note: (**A**) *Calathea lutea* (Aubl.) Schult. (bijao) (identification code: 4770, Herbarium QUPS, Ecuador); (**B**) *Calathea inocephala* (Kuntze) H. Kenn. & Nicolson (shutupipanga) (identification code: 4795, Herbarium QUPS, Ecuador); (**C**) chicken broth maito; and (**D**) fish maito.

Fully developed leaves of *Calathea lutea* (Figure 1A) and *Calathea inocephala* (Figure 1B) were randomly collected from the same representative area of the Ecuadorian Amazon (1°44′21″ S, 77° 29′1″ W) during March 2020. A portion of the plant material was used for botanical identification in the herbarium of the Universidad Politécnica Salesiana of Quito, Ecuador (QUPS, Ecuador), while the rest was divided into two fractions. The first fresh fraction of the sample was subjected to physicochemical analysis, and the other was stored frozen (−80 °C) before being freeze-dried by a Christ Alpha 1-4 LDplus equipment (GmbH, Osterode am Harz, Germany) to determine bioactive compounds and evaluate biological activities.

The physicochemical parameters included pH measurement using a SevenMultiS47 digital potentiometer (Mettler Toledo, Columbus, OH, USA), soluble solids measurement with a Hitech RHB-32 refractometer (G-Won Hitech Co., Ltd., Seoul, Republic of Korea), moisture content determination by drying at 110 °C in a Memmert Be 20 oven (Memmert GmbH + Co.KG, Barcelona, Spain), and ash quantification by incineration at 550 °C in a muffle furnace (Thermo Fisher Scientific, Waltham, MA, USA) [9].

For mineral analysis (Ca, Fe, Na, K, and P), 40 mg of lyophilised leaves were subjected to acid digestion with 5 mL of concentrated nitric acid in an Xpert microwave system (Berghof products + Instruments GmbH, Eningen unter Achalm, Germany) under controlled temperature and pressure conditions. The programme initiated with a gradual increase to 140 °C at 30 bar using 70% of the maximum microwave output for 5 min. This was followed by an intensified digestion step at 200 °C and 35 bar with 80% power sustained for 15 min to ensure complete matrix decomposition. The cycle concluded with a passive cooling phase at 50 °C and 25 bar, during which microwave energy was discontinued for 10 min to allow thermal and pressure equilibration. Subsequently, the digested solutions were diluted to a final volume of 25 mL with deionised water, and the elements were quantified by atomic spectrometry using a Varian Spectra AA-55 device (Agilent Technologies, Santa Clara, CA, USA) [9].

The qualitative identification of secondary metabolites involved the detection of alkaloids, acetogenins, anthraquinones, flavonoids, phenolic compounds, saponins, steroids, tannins, and terpenes, as described by León-Fernández et al. [10]. To obtain the extract, 20 mg of lyophilised leaf material was transferred to a microcentrifuge tube and resuspended in 1 mL of deionised water. The mixture was subjected to mechanical homogenisation followed by ultrasonic-assisted extraction using an FS60 ultrasonic bath (Fisher Scientific Inc., Waltham, MA, USA) for 3 min to enhance compound dispersion and extraction efficiency. Subsequently, the mixture was centrifuged in an Eppendorf 5430 (Eppendorf AG, Hamburg, Germany) at 14,000 rpm for 5 min at 4 °C to achieve phase separation. The supernatant was carefully collected, and the residual solid was subsequently subjected to two additional extraction steps using 500 µL of deionised water to enhance compound recovery. The combined extracts were used to qualitatively detect different groups of secondary metabolites.

### 2.3. Bioactive Constituents

#### 2.3.1. Vitamin C Identification

Vitamin C (ascorbic acid) was determined following an acid-stabilised extraction protocol. Briefly, 40 mg of lyophilised leaf material was accurately weighed and extracted with 1200 µL of 3% (*w*/*v*) metaphosphoric acid, to which 200 µL of 0.2% *DL*-homocysteine was added as a reducing agent to prevent oxidative degradation of the analyte. The mixture was subjected to ultrasonic treatment for 1 min to enhance extraction efficiency and subsequently adjusted to a final volume of 2 mL with deionised water. The suspension was centrifuged at 14,000 rpm for 5 min at 4 °C to separate the supernatant, which was carefully collected and filtered through a 0.45 µm membrane filter (24 mm diameter, DPVF) prior to chromatographic analysis. All extractions were performed in triplicate to ensure analytical reproducibility.

Chromatographic analysis was carried out using an Agilent RRLC 1200 high-performance liquid chromatography system (Agilent Technologies, Santa Clara, CA, USA) equipped with a diode-array detector (DAD-UV-Vis) operating at 244 nm. Separation was achieved on a Zorbax Eclipse XDB-C18 column (80 Å pore size, 4.6 × 50 mm, 1.8 µm) (Agilent Technologies, Santa Clara, CA, USA) under isocratic conditions. The mobile phase consisted of an aqueous solution containing 1.5% monobasic potassium phosphate and 1.8% n-acetyl-n,n,n-trimethylammonium bromide mixed in a 90:10 (*v*/*v*) proportion, delivered at a constant flow rate of 1.0 mL/min. Samples were injected in duplicate to verify chromatographic repeatability. Data acquisition and peak integration were performed using ChemStation software (version 2.15.26). Compound identification was established through comparison of retention time and UV-Vis spectral characteristics with those of an authentic standard, with additional confirmation attained using an internal standard approach. Quantification was based on an external calibration curve prepared from a 1 mg/mL *L*-(+)-ascorbic acid stock solution. Serial injections ranging from 1 to 20 µL were used to construct the linear regression model (R^2^ of 0.99). The method exhibited limits of detection (LODs) and quantification (LOQs) of 0.20 ppm and 0.65 ppm, respectively. Final concentrations were expressed as milligrams of vitamin C per 100 g of dry leaf weight (mg/100 g DW) [11].

#### 2.3.2. Organic Acid Identification

Organic acids (citric, malic, and tartaric acid) were quantified by weighing 20 mg of freeze-dried leaves, which were mixed with 1500 µL of 0.02 N sulfuric acid supplemented with 0.02% *DL*-homocysteine and 0.05% metaphosphoric acid. After ultrasonic treatment for three minutes, the extract was adjusted to a final volume of 2 mL with deionised water. The extract was centrifuged at 14,000 rpm for 5 min at 4 °C to facilitate phase separation, and the supernatant was carefully collected. Prior to chromatographic analysis, the extract was filtered through a 0.45 µm filter (24 mm diameter DPVF). All extractions were conducted in triplicate to ensure analytical reproducibility.

Organic acids were determined using an Agilent RRLC 1200 high-performance liquid chromatography system equipped with a diode-array detector (DAD-UV-Vis) operating at 210 nm. Separation was achieved on a YMC-Triart C18 column (120 A pore size, 150 × 4.6 mm, 3 µm) (YMC Europe GmbH, Dinslaken, Germany) under isocratic conditions. The mobile phase consisted of 0.027% sulfuric acid in water, delivered at a constant flow rate of 1.0 mL/min. Samples were injected in duplicate to verify chromatographic repeatability, and data acquisition and integration were performed using ChemStation software (version 2.15.26). Compound identification was based on comparison of retention times and UV absorption spectra with those of authenticated standards, supported by internal standard verification when applicable. External calibration curves were prepared individually for *L*-(+)-tartaric, citric, and malic acids from 100 mg/mL stock solutions. Injection volumes ranging from 1 to 20 µL were used to generate linear regression models (R^2^ of 0.99). The method demonstrated limits of detection (LODs) and quantification (LOQs) of 0.06 and 0.17 ppm for tartaric acid, 0.08 and 0.26 ppm for citric acid, and 0.13 and 0.39 ppm for malic acid, respectively. The results were expressed as milligrams of organic acid per 100 g of dry leaf weight (mg/100 g DW) [11].

#### 2.3.3. Carotenoid Identification

Carotenoids were quantified by weighing 20 mg of freeze-dried leaves, which were mixed with 250 µL of methanol, 250 µL of acetone, and 500 µL of dichloromethane. The mixture was sonicated for 2 min, and the supernatant was recovered by centrifugation. The solid residue was re-extracted with 500 µL of the mixture, repeating the procedure as many times as necessary until the pigment was extracted entirely. The coloured solution obtained was concentrated to dryness by rotary evaporation in a Buchi TM R-100 apparatus (Fisher Scientific, Hampton, NH, USA) at a temperature not exceeding 40 °C under reduced pressure. The dry residue was redissolved in 40 µL of ethyl acetate, and the resulting supernatant was recovered by centrifugation before being transferred to a vial for the liquid chromatography analysis. The extraction of carotenoids was carried out in triplicate.

Carotenoid separation was carried out using an Agilent RRLC 1200 high-performance liquid chromatography system coupled to a diode-array detector (DAD-UV-Vis) operating within the 350–450 nm spectral range. Chromatographic resolution was achieved on a YMC C30 column (3 µm particle size, 4.6 × 150 mm) (YMC Europe GmbH, Dinslaken, Germany), specifically selected for its enhanced selectivity toward structurally related carotenoids. The mobile phase consisted of methanol (A), methyl tert-butyl ether (B), and water (C), delivered at a constant flow rate of 1.0 mL/min under a programmed gradient elution. The gradient was 95% A + 5% B + 0% C at 0 min; 95% A + 5% B + 0% C at 5 min; 95% A+ 5% B + 0% C at 5 min; 89% A + 11% B + 10% C at 10 min; 89% A + 11% B + 0% C at 10 min; 75% A + 25% B + 0% C at 16 min; 40% A + 60% B + 0% C at 20 min; 15% A + 85% B + 0% C at 22 min; 90% A + 5% B + 5% C at 25 min; and 90% A + 5% B + 5% C at 28 min.

Each sample was injected in duplicate to ensure the reproducibility of the chromatographic response. The chromatograms were processed using ChemStation software (version 2.15.26). The identification and quantification of carotenoids were based on comparisons of retention times, spectra at 350 nm or 450 nm, and the internal standard. The calibration curve was prepared from a 1 mg/mL standard solution of astaxanthin, lutein, violaxanthin, zeaxanthin, trans-β-apo-8-carotenal, α-carotene, β-carotene, zeinoxanthin, β-cryptoxanthin, and lycopene, and different volumes (1 to 20 µL) were injected to establish a linear relationship with an R^2^ of 0.99. LOD and LOQ values for the most important carotenoids were 0.007 and 0.02 ppm for lutein, 0.029 and 0.09 ppm for β-carotene, and 0.003 and 0.008 ppm for zeaxanthin, respectively. The results were expressed as milligrams of carotenoid per 100 g of dry weight of leaves (mg/100 g DW). The total carotenoids under study correspond to the sum of all the major individual compounds detected [9].

#### 2.3.4. Phenolic Compound Identification

Phenolic compounds were extracted using an acidified methanolic protocol. Briefly, 20 mg of lyophilised leaf material was accurately weighed and combined with 1000 µL of 80% methanol containing 0.1% (*v*/*v*) HCl to enhance the solubility and stabilisation of phenolic constituents. The mixture was subjected to ultrasonic-assisted extraction for 3 min, followed by centrifugation to separate the supernatant. The remaining solid residue was re-extracted twice with 500 µL of the same solvent system to maximise recovery. The extract was filtered through a 0.45 µm filter (24 mm diameter, DPVF) prior to chromatographic analysis. All extractions were performed in triplicate to ensure analytical reproducibility.

Chromatographic analysis was conducted using an Agilent RRLC 1200 system equipped with a diode-array detector (DAD-UV-Vis) operating within the 280 and 370 nm wavelength range. Separation was achieved on a Zorbax Eclipse Plus C18 column (4.6 × 150 mm, 5 µm) (Agilent Technologies, USA). Elution was performed using a binary gradient system consisting of 0.01% formic acid in water (solvent A) and acetonitrile (solvent B), delivered at a constant flow rate of 1.0 mL/min. The gradient programme initiated with 100% A at time zero, transitioned to 95% A and 5% B at 5 min, and progressively reached 50% A and 50% B at 20 min, followed by a column washing and re-equilibration step prior to subsequent injections.

Each sample was injected in duplicate to ensure the reproducibility of the chromatographic response. The chromatograms were processed using ChemStation software (version 2.15.26). The identification and quantification of phenolics were based on comparisons of retention times, spectra at 280 nm, 320 nm, and 370 nm, and the internal standard. The calibration curve was prepared from a 1 mg/mL standard solution of caffeic acid, chlorogenic acid, chrysin, ferulic acid, gallic acid, 2,5-dihydroxibenzoic acid, 3-hydroxybenzoic acid, kaempferol, luteolin, *m*-coumaric acid, naringin, *o*-coumaric acid, *p*-coumaric acid, *p*-hydroxybenzoic acid, quercetin, shikimic acid, syringic acid, and vanillic acid, and different volumes (1 to 20 µL) were injected to establish the linear relationship with an R^2^ of 0.99. LOD and LOQ for the most important phenolics were 0.009 ppm and 0.028 ppm for chlorogenic acid, 0.048 ppm and 0.145 ppm for caffeic acid, 0.007 ppm and 0.021 ppm for gallic acid, respectively. The results were expressed as milligrams of phenolics per 100 g of dry weight of leaves (mg/100 g DW). The total phenolics under study correspond to the sum of all the major individual compounds detected [9].

### 2.4. Antimicrobial Activity

#### 2.4.1. Preparation of the Freeze-Dried Extract

An enriched phenolic extract was obtained using a hydroethanolic extraction procedure. Briefly, 2 g of lyophilised leaf material was accurately weighed and extracted with 25 mL of 50% ethanol (*v*/*v*). The suspension was subjected to ultrasonic-assisted extraction for 6 min to promote solvent penetration and metabolite release. Following extraction, the mixture was centrifuged using a microcentrifuge (Eppendorf, Bochum, Germany), and the supernatant was carefully collected. The solid residue was subsequently re-extracted twice under identical conditions to ensure maximal recovery of phenolic constituents. The combined extracts were filtered through Whatman No. 1 filter paper and concentrated under reduced pressure using a rotary evaporator at a temperature below 40 °C to prevent thermal degradation. The resulting concentrate was frozen and lyophilised to obtain a dry extract, which was stored at a low temperature until further analysis.

#### 2.4.2. Antibacterial Activity

The resulting dry extract was reconstituted in 1 mL of sterile distilled water to evaluate antimicrobial activity using the well-diffusion and microdilution methods, conducted in accordance with Clinical and Laboratory Standards Institute (CLSI) guidelines, with minor methodological adaptations. Antibacterial activity was tested against reference strains *Staphylococcus aureus* ATCC 6538P, *Escherichia coli* ATCC 8739, *Pseudomonas aeruginosa* ATCC 9027, and *Streptococcus mutans* ATCC 25175. Each microorganism was cultured in a brain heart infusion (BHI) broth and incubated aerobically at 37 °C for 48 h. Following incubation, bacterial suspensions were standardised to 0.5 McFarland turbidity (approximately 1.5 × 10^8^ CFU/mL) and uniformly spread onto Mueller–Hinton agar plates. Wells (6 mm diameter) were aseptically bored into the agar using a sterile tip, and 80 µL of reconstituted extracts was dispensed into each well. Plates were incubated at 35 °C for 48 h under aerobic conditions. Streptomycin served as the positive control, while sterile distilled water was used as the negative control. Antimicrobial efficacy was evaluated by measuring the diameter of the inhibition halos (mm) surrounding each well [12].

Additionally, the minimal inhibitory concentration (MIC) was determined following CLSI guidelines. Extracts (300 mg/mL) and bacterial inocula (adjusted to 0.5 McFarland) were tested in triplicate using 96-well microplates. Serial dilutions of the extract were prepared in Muller–Hinton broth in the microplate, with a final volume of 200 μL per well. Each well received 20 μL of the microbial inoculum. There were growth controls (culture medium + microorganisms), a positive control (antibiotic + microorganism), a sterility control (culture medium only), and a vehicle (culture medium + sterile water). Incubation was carried out at 37 °C for 24 h to allow bacterial growth, and antimicrobial activity was subsequently determined.

After the incubation period, 20 μL of 4% TTC was added to all wells used to determine the MIC. The microplate was sealed with aluminium foil and incubated at 37 °C for 2 h, after which a colour change was observed, interpreted as the bacterium’s presence. Based on this, the last well that presented this colouration was identified. This would represent the MIC of the extract towards the strain inoculated into the microplate.

#### 2.4.3. Antibacterial Activity in Multi-Resistant Bacteria

The antibacterial activity of the lyophilised ethanolic extract of ‘shutupipanga’ was evaluated against seven multidrug-resistant (MDR) clinical isolates, such as *Klebsiella pneumoniae*, *Escherichia coli*, *Salmonella enterica serovar Kentucky*, *Enterococcus faecalis*, *Staphylococcus epidermidis*, *Enterococcus faecium*, and *Pseudomonas aeruginosa.* All strains were provided by the National Institute of Public Health Research of Ecuador (INSPI) and form part of its External Quality Assessment Programme.

Bacterial suspensions were prepared in brain heart infusion (BHI) broth and adjusted to a final concentration of 5 × 10^5^ CFU/mL. The freeze-dried ethanolic extract was dissolved in dimethyl sulfoxide (DMSO) to obtain a stock solution at 320 mg/mL. Nourseothricin (100 µg/mL) was included as a positive antimicrobial control. Wells containing BHI alone and BHI supplemented with the extract at the corresponding concentrations served as sterility and solvent controls, respectively. Antibacterial activity was determined using the broth microdilution method, performed according to CLSI recommendations with minor adaptations [13]. Briefly, 5 µL of the extract stock solution was added to 195 µL of the standardised bacterial inoculum (5 × 10^5^ CFU/mL), resulting in a final assay volume of 200 µL per well. Microplates were incubated at 37 °C for 20 h under continuous orbital agitation (300 rpm, double orbital mode). Optical density at 600 nm (OD_600_) was recorded at time zero and after 24 h of incubation to monitor bacterial growth. The minimum inhibitory concentration (MIC) was defined as the lowest extract concentration that completely suppressed visible bacterial growth, as determined by OD_600_ measurements. All assays were conducted in at least three independent replicates to ensure reproducibility.

#### 2.4.4. Antifungal Activity

Antifungal activity was assessed against *Candida albicans* ATCC 1031 and *Candida tropicalis* CC 13803. Yeast strains were propagated in yeast peptone dextrose (YPD) broth and incubated aerobically at 30 °C for 48 h. Following incubation, cell suspensions were standardised to a 0.5 McFarland turbidity (approximately 5 × 10^5^ CFU/mL). Aliquots of the adjusted suspensions were uniformly spread onto Sabouraud dextrose agar plates. Agar wells (6 mm diameter) were aseptically prepared, and 80 µL of the test extracts was dispensed into each well. The plates were subsequently incubated at 35 °C for 48 h under aerobic conditions. Fluconazole was employed as the positive antifungal control, whereas sterile distilled water served as the negative control. Antifungal efficacy was determined by measuring the diameter of the inhibition zones (mm) surrounding each well [14].

Additionally, the minimal inhibitory concentration (MIC) was determined with extracts (300 mg/L) and yeast inocula (adjusted to 5 × 10^5^ CFU/mL) in triplicate using 96-well microplates. Serial dilutions of the extract were prepared in yeast extract peptone dextrose broth in the microplate, with a final volume of 200 μL per well. Each well received 20 μL of yeast suspension. There were growth controls (culture medium + yeast suspension), a positive control (antibiotic + yeast suspension), a sterility control (culture medium only), and a vehicle (culture medium + sterile water). Plates were incubated at 37 °C for 24 h to assess antifungal activity.

After the incubation period, 20 μL of 4% TTC was added to all wells used to determine the MIC. The microplate was sealed with aluminium foil and incubated at 37 °C for 2 h, after which a colour change was observed, interpreted as the bacterium’s presence. Based on this, the last well that presented this colouration was identified as the MIC of the extract towards the yeast inoculated into the microplate.

### 2.5. Antioxidant Activity

The antioxidant activity of the freeze-dried leaf extracts was evaluated using the ABTS and DPPH methods. To prepare the extract, 20 mg of freeze-dried leaves was weighed and mixed with 2 mL of methanol. The suspension was sonicated for 3 min in an ultrasonic bath, and the supernatant was recovered by centrifugation. All extractions were performed in triplicate [11].

For the formation of the DPPH radical, 10 mg of the reagent was dissolved in 50 mL of methanol, and the solution was adjusted to 0.7 ± 0.02 at 515 nm. For the assay, 20 µL of the extract was mixed with 280 µL of the DPPH radical solution in a 96-well VMT microplate. The mixture was incubated with agitation for 30 min at room temperature in the dark on the Shaker 4310 orbital platform (Fisher Scientific, Waltham, MA, USA) before absorbance was measured in a BioTek H1 spectrophotometer (Agilent Scientific Instruments, Santa Clara, CA, USA).

In the ABTS method, the radical solution was prepared by mixing 7 mM ABTS with 0.45 nM potassium persulfate, allowing it to stand for 16 h to form the ABTS^+^ radical, and adjusting the absorbance to 0.7 ± 0.02 at 734 nm. For the analysis, 20 µL of the extract was combined with 280 µL of the ABTS^+^ solution, kept in the dark, and stirred constantly before reading.

In both methods, antioxidant activity was determined using calibration curves with 0.99 R^2^ prepared with Trolox at 10 mM, ranging from 0.4 to 4 mM in DPPH and from 0.2 to 0.7 mM in methanol for ABTS. Readings were taken in duplicate, and the results were expressed as millimoles of Trolox equivalent per 100 g of dry weight (mmol TE/100 g DW).

### 2.6. Biofilm Inhibition Activity

Given the presence of bioactive compounds in the species analysed, *Calathea inocephala* was selected for this study because it had the highest concentration. The biofilm inhibition potential of *C. inocephala* dry extract (Section 2.4.1) was assessed against selected biofilm-forming microorganisms, including *S. aureus* ATCC 25923, *L. monocytogenes* ATCC 13932, *B. cepacia* ATCC 25416, and the fungal strain *C. tropicalis* ATCC 13803. All strains were cultured overnight in tryptic soy broth supplemented with 1% glucose (TSB+G) at 37 °C [15].

Briefly, the overnight cultures were diluted 1:100 in fresh medium and plated along with a range of plant extract concentrations (5 mg/mL–1 µg/mL). A total volume of 150 µL of each suspension was transferred into 96-well plates and incubated statically at 37 °C for 24 h. Following incubation, planktonic cells were removed by aspiration and washed with phosphate-buffered saline (PBS, 1×, pH 7.2). The plates were dried in a laboratory oven at 60 °C for one hour. Biofilms were stained with 150 µL of 0.1% (*w*/*v*) crystal violet solution for 20 min at room temperature, then washed 3 times with PBS. The retained stain was solubilised with 150 µL of 96% ethanol for 30 min, and biofilm biomass was quantified spectrophotometrically at 570 nm. The data were analysed using the following formula:

Inhibitory rate (%) = [(Positive control OD_570_ nm − Sample OD_570_ nm)/Positive control OD_570_ nm] × 100

### 2.7. Haemolytic Activity

Given the presence of bioactive compounds in the species analysed, *Calathea inocephala* was selected for this study because it had the highest concentration. The haemolytic activity of the *C. inocepha* dry extract (Section 2.4.1) was evaluated using a modification from Sæbø et al., designed to detect erythrocyte membrane disruption [16]. Fresh, defibrinated sheep blood (10 mL) was subjected to triple washing in phosphate-buffered saline (PBS, 1×, pH 7.4) via centrifugation at 1700× *g* for 5 min. The packed erythrocytes were then resuspended to achieve a final 1% (*v*/*v*) cell suspension in PBS.

For the assay, equal volumes of the erythrocyte suspension and either the extract solution or the control were mixed in polypropylene 96-well plates. The *C. inocepha* extract was tested at five concentrations: 2500, 1250, 625, 312.5, and 156.25 μg/mL. Triton X-100 (Merck, Darmstadt, Germany) (10% *v*/*v*) was used as a positive control to induce complete haemolysis, while PBS 1× served as the negative control. The mixtures were incubated at 37 °C for 1 h under gentle agitation to simulate physiological conditions and facilitate interaction between the extract and the erythrocyte membranes.

Following incubation, the plates were centrifuged at 1700× *g* for 5 min to pellet intact cells and debris. The resulting supernatants—containing any haemoglobin released due to membrane rupture—were carefully transferred to flat-bottom, transparent 96-well plates for absorbance analysis.

To accurately monitor haemolysis and account for potential interference from plant pigments, full absorbance spectra (340–800 nm, 10 nm intervals) were recorded using a Cytation5 multi-mode plate reader (BioTek Instruments, Winooski, VT, USA). Parallel colour controls, consisting of extract dilutions without erythrocytes, were included to quantify and subtract background absorbance arising from the extract’s intrinsic colouration, ensuring reliable discrimination of haemolysis-derived signals.

Each condition was analysed in triplicate, and the experiment was independently replicated three times. To quantify the degree of haemolysis, a composite absorbance value (OD_pond) was calculated by applying a weighted average of absorbance at 410 nm (0.6), 540 nm (0.2), and 580 nm (0.2), which correspond to the major absorbance peaks of oxyhaemoglobin [17]. This weighting scheme emphasises the diagnostic Soret band while incorporating contributions from secondary absorption features, improving signal stability and analytical precision.

The percentage of haemolysis (%HR) was determined for each sample using the following equation:

(1) $$\%HR=\frac{\left(ODsamplepond-ODnegpond\right)}{\left(ODpospond-ODnegpond\right)}\times 100$$

### 2.8. Statistical Analysis

Statistical analyses were conducted using RStudio (version 4.4.1), Statgraphics Centurion XVII, and SigmaPlot 14.0 software. Data are presented as mean ± standard deviation (SD). Normality and homogeneity of variance were verified prior to inferential testing. Differences among groups were evaluated by one-way analysis of variance (ANOVA), and multiple comparisons were performed using Tukey’s honestly significant difference (HSD) test. Statistical significance was established at *p* < 0.05. Furthermore, Pearson’s correlation coefficients were calculated at a 95% confidence level to explore linear associations between the analysed variables.

## 3. Results and Discussion

### 3.1. Physicochemical Properties

Table 1 shows the physicochemical parameters of *Calathea lutea* and *Calathea inocephala* leaves. The physicochemical characterisation showed differences between the two species under study. The pH values indicate that *C. lutea* is slightly more neutral than *C. inocephala*, reflecting a higher buffering capacity that could influence its suitability for food wrapping or traditional preparations where mild acidity may affect flavour or preservation. Likewise, the low soluble solids values in both species confirm limited accumulation of soluble carbohydrates in the leaves, which is consistent with their morphological function being more structural than storage-related [18].

The total titratable acidity content also showed significant differences between the two species studied. The moisture content differed significantly between the two species. Such differences could be explained by variations in leaf anatomy (e.g., mesophyll thickness, stomatal density, water capacitance) that affect water accumulation in the tissue. Recent research has shown that leaf water content directly influences other functional traits, such as leaf area, photosynthesis, and leaf-to-mass ratio [19]. Thus, the higher moisture content of *C. inocephala* could favour greater leaf flexibility or reduced fragility, with implications for its practical use in food wrapping or preparation.

The ash percentage was higher in *C. lutea* than in *C. inocephala*, indicating a higher total mineral content in *C. lutea*. This also suggests differences in foliar mineral accumulation that could be due to variations in soil availability, absorption efficiency, or internal mineral accumulation [20].

Regarding the mineral profile, the data show that *C. inocephala* accumulated significantly more calcium than *C. lutea*. *C. lutea*, on the other hand, showed higher iron and magnesium levels than *C. inocephala*. These differences suggest that each species has a distinct mineral accumulation pattern, likely in response to ecological, physiological, or genetic conditions. In this regard, recent reviews of plant nutrition in changing environments indicate that the uptake, accumulation, and distribution of minerals in leaves are strongly modulated by abiotic stress factors (such as water availability, soil, and nutrient availability) and are linked to key physiological functions [21]. For example, greater calcium availability may contribute to increased rigidity or structural support in leaf tissue. At the same time, high iron and magnesium values reflect a high metabolic demand for chlorophyll and active enzymes [22].

Potassium and sodium also differed. A higher K/Na ratio in *C. lutea* could indicate a better ionic balance for certain metabolic and osmotic functions in the leaf. Thus, the results suggest that *C. lutea* is distinguished by a higher total mineral content, particularly in Fe, Mg, and K, which could make it more attractive from a nutritional or functional perspective, while *C. inocephala* has higher leaf moisture and Ca content, which may confer structural and handling advantages [22].

Table 2 shows the presence or absence of secondary metabolites in the freeze-dried leaves of *Calathea lutea* and *Calathea inocephala.* Qualitative screening showed that both species are positive for phenols, tannins, and acetogenins, while *C. inocephala* also contains steroids (phytosterols), terpenoids, and flavonoids; however, both were negative for alkaloids, anthraquinones, and saponins. This pattern suggests defensive and photoprotective strategies that, in *C. inocephala*, rely more on flavonoids/terpenoids/phytosterols and on polyphenols in both.

The concurrent presence of phenols and tannins indicates strong antioxidant and antimicrobial potential in leaf tissues, as these molecules are recognised for their ability to quench free radicals and bind redox-active metal ions, chelate transition metals, and disrupt microbial cell membranes and enzymatic activity [23]. The exclusive detection of flavonoids in *C. inocephala* suggests a higher investment in photoprotection and tolerance to oxidative and ultraviolet stress, aligning with the established role of flavonoids as UV filters and redox modulators in plant leaves [24]. Likewise, the presence of phytosterols in *C. inocephala* suggests the formation of stable lipid microdomains and maintenance of membrane integrity, which may enhance plant defence and resilience under stress conditions [25]. Acetogenins, characteristic metabolites of the Annonaceae family, exhibit recognised anticarcinogenic properties [11], emphasising the importance of further investigations to corroborate this qualitative finding.

### 3.2. Bioactive Constituents

**Figure 2:**
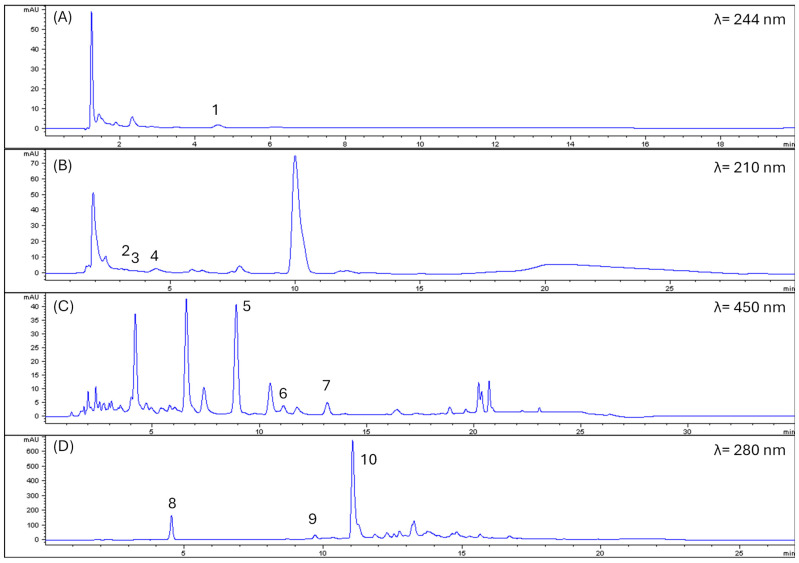
Examples of chromatograms of vitamin C (**A**) and carotenoids (**C**) in bijao; organic acid (**B**) and phenolics in shutupipanga (**D**). Note: 1, vitamin C; 2, tartaric acid; 3, malic acid; 4, citric acid; 5, lutein; 6, zeaxanthin; 7, zeinoxanthin; 8, gallic acid; 9, chlorogenic acid; 10, caffeic acid.

Table 3 shows the results of the quantification of bioactive compounds, including vitamin C, organic acids (citric, malic, and tartaric acids), carotenoid profile, and phenolic compound profile. In turn, Figure 2 shows the chromatograms of vitamin C, organic acids, carotenoids at 450 nm, and phenolic compounds at 280 nm.

The vitamin C content was higher in *C. lutea* than in *C. inocephala*. This result could be attributed to the photosynthetic metabolism of each species, where ascorbate levels adjust to environmental stress or light intensity. It has been shown that the concentration of vitamin C in leaf tissues depends on the activity of the ascorbate–glutathione cycle and exposure to solar radiation [26].

In terms of organic acids, *C. lutea* had a significantly higher total concentration than *C. inocephala*, with a predominance of tartaric acid. This profile suggests a greater accumulation of Krebs cycle metabolites, which could reflect greater metabolic and photosynthetic activity. Citric and malic acids, in addition to their energetic role, act as chelating agents and antioxidants in plant tissues [27].

The carotenoid profile showed a clear divergence among the species studied. *C. inocephala* accumulated a higher concentration of carotenoids compared to *C. lutea*, whose accumulation was minimal. This contrast indicates a greater biosynthetic capacity for pigments in *C. inocephala*, especially lutein and β-carotene, which are essential for photoprotection and chloroplast stability. Several studies have shown that differential carotenoid accumulation across species is related to light exposure, tissue age, and genetic regulation of the isoprenoid pathway [28,29].

The concentration of total chlorophylls was considerably higher in C. *inocephala* than in *C. lutea*. This finding could be explained by differences in parenchyma structure and chloroplast density, which translate into greater photosynthetic efficiency. The presence of pheophytins in both species indicates partial chlorophyll degradation processes, possibly associated with environmental stress or tissue maturity [30].

The phenolic profile showed the most marked difference between species. *C. inocephala* had a total phenol concentration almost ten times higher than that of *C. lutea*. In particular, the levels of caffeic acid and kaempferol stood out; these are compounds known for their high antioxidant capacity and for their role in plant defence against oxidative stress. This notable difference suggests a metabolic specialisation of *C. inocephala* towards the synthesis of phenolic compounds, possibly induced by environmental factors, such as UV radiation typical of the Amazon region [31].

The antioxidant activity results indicate very similar values between both species, although *Calathea inocephala* showed a slight superiority in both the ABTS and DPPH methods compared to *Calathea lutea*. These results are consistent with the fact that antioxidant capacity in leaves is usually strongly correlated with total polyphenols and flavonoids measured in the extract. Recent studies report a high correlation between total polyphenols and ABTS/DPPH in plant matrices, supporting the idea that small differences in phenols/flavonoids explain the advantage of *C. inocephala* [32].

The differences between DPPH and ABTS are due to their mechanisms and reaction kinetics (sensitivity to compounds of different polarity and sensitivity to different compounds), so they do not always coincide in absolute magnitude; however, both are valid for comparing profiles when the test conditions are controlled [33].

### 3.3. Antimicrobial Activity

#### 3.3.1. Antibacterial and Antifungal Activity with ATCC Microorganisms

Table 4 shows the inhibition zone values of the dry extracts of *Calathea lutea* and *Calathea inocephala* against Gram-negative bacteria (*Escherichia coli*, *Pseudomonas aeruginosa*), Gram-positive bacteria (*Staphylococcus aureus*, *Streptococcus mutans*), and yeasts (*Candida albicans*, *Candida tropicalis*). In *Calathea lutea*, the inhibition zones were similar for *E. coli* and *S. aureus* and smaller for *P. aeruginosa* and yeasts. In *Calathea inocephala*, greater inhibition was observed against *S. aureus* and *S. mutans*, moderate inhibition against *E. coli*, and greater inhibition against *C. tropicalis*.

The results suggest that *C. inocephala* has a broader and more potent antimicrobial profile than *C. lutea* in the strains analysed, which may be due to higher concentrations or greater diversity of bioactive compounds (e.g., phenols, flavonoids, and terpenoids) and greater diffusion efficiency in the agar medium. In this context, the literature recognises that plant extracts with high phenolic and flavonoid content (including tannins, flavones, and flavanols) exhibit strong inhibition zones against Gram-positive and Gram-negative bacteria when tested by diffusion methods. For example, a recent review documents standard mechanisms, such as alteration of microbial membrane permeability, inhibition of cell wall synthesis enzymes, and chelation of metal ions essential to the pathogen [34].

Additionally, the literature indicates that the size of the inhibition zone correlates with the concentration of extracted secondary metabolites, the extract’s diffusion in the agar, and the strain’s sensitivity. Recent studies show that extracts with zones of 15–25 mm already exhibit significant activity, providing a basis for further evaluation [35]. However, moderate activity against fungi (*C. albicans*, *C. tropicalis*) also confirms that the extracts have antifungal activity, although this is less than their antibacterial activity, consistent with studies reporting that secondary metabolites must be at higher concentrations or require synergy to achieve robust antifungal effects [36].

Table 5 shows the minimum inhibitory concentration (MIC) of *Calathea lutea* and *Calathea inocephala* against Gram-negative bacteria (*Escherichia coli* and *Pseudomonas aeruginosa*), Gram-positive bacteria (*Staphylococcus aureus* and *Streptococcus mutans*), and yeasts (*Candida albicans* and *Candida tropicalis*). *C. lutea* had a higher MIC compared to *C. inocephala.* This difference could be due to the presence of phenolic acids and tannins. These compounds can alter the permeability of the outer membrane, which is rich in lipopolysaccharides and is characteristic of Gram-negative bacteria [23]. In contrast, *C. inocephala* was more effective against *S. aureus* than *C. lutea*. The higher concentration of flavonoids and terpenoids can explain this behaviour. These compounds act on Gram-positive bacteria by destabilising the cell wall and blocking the synthesis of essential proteins [37]. In *S. mutans*, both extracts exhibited moderate activity, likely due to phenolic acids such as caffeic and chlorogenic acids, previously reported to inhibit oral biofilm formation and bacterial glucosyltransferase [32].

#### 3.3.2. Antibacterial Activity in Multi-Resistant Bacteria

Table 6 shows the minimal inhibitory concentration of *C. inocephala* freeze-dried extract. This exhibited activity against three of the seven multidrug-resistant bacteria tested, *E. faecium*, *E. faecalis*, and *S. epidermidis*. These last two have the lowest MIC (1.00 mg/mL) among the other strains. The greater sensitivity of Gram-positive bacteria can be attributed to the more permeable structure of their cell wall, composed mainly of peptidoglycan, which allows better penetration of phenolic compounds and flavonoids present in the extract. Conversely, Gram-negative bacteria are characterised by an additional outer membrane composed of lipopolysaccharides, which reduces membrane permeability and impedes the uptake of polar bioactive molecules [36].

### 3.4. Biofilm Inhibition Activity

**Figure 3:**
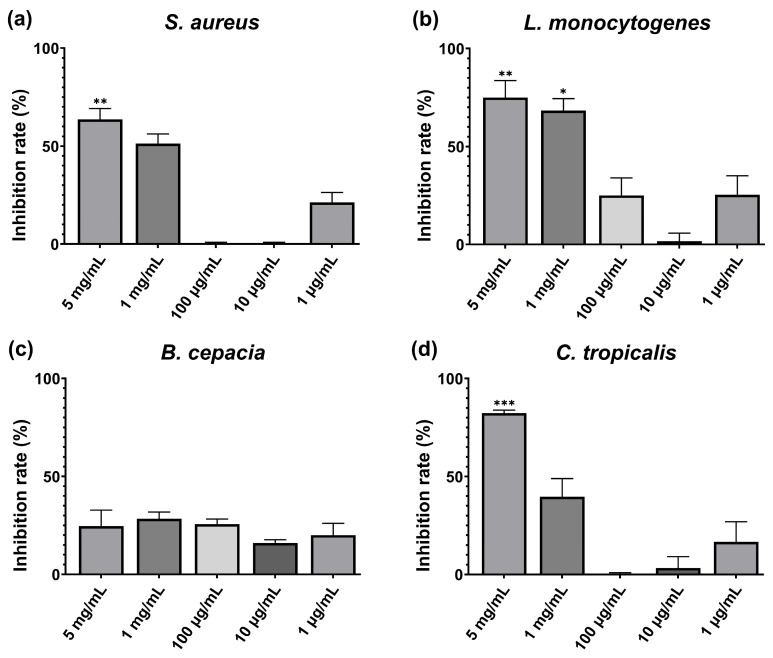
Evaluation of biofilm inhibition IC50 (MBIC50) of (**a**) *Staphylococcus aureus* ATCC 25923, (**b**) *Listeria monocytogenes* ATCC 13932, (**c**) *Burkholderia cepacia* ATCC 25, and (**d**) *Candida tropicalis* ATCC 13,803 after 24 h incubation with *C. inocephala* extract at different concentrations (5 mg/mL–1 µg/mL). Treatments at different concentrations were compared with a 50% theoretical inhibition standard to assess statistical significance using a two-way ANOVA. All the values are mean ± SD, *p*-value (*) < 0.05, (**) < 0.01, (***) < 0.001.

The biofilm-inhibition activity of the *C. inocephala* freeze-dried extract was evaluated against biofilm-forming microorganisms, including *Staphylococcus aureus* ATCC 25923, *Listeria monocytogenes* ATCC 13932, *Burkholderia cepacia* ATCC 25, and *Candida tropicalis*. The resulting MBIC50 (minimum biofilm-inhibiting concentration for 50% inhibition) is shown in Figure 3. The extract displayed statistically significant inhibitory activity against three of the four strains used during this evaluation. The lowest BMIC50 value was observed for *L. monocytogenes* at 1 mg/mL, with 65% inhibition (*p*-value < 0.05). Additionally, both *S. aureus* and *C. tropicalis* were significantly inhibited at 5 mg/mL (63% and 82%, respectively). The effectiveness of the dry extract of *Calathea inocephala* against Listeria monocytogenes is attributed to the high concentration of caffeic acid in this species. This phenolic compound has been shown to interfere with bacterial cell wall synthesis, a process essential for biofilm formation and stability. Altering this structure can prevent bacterial adhesion to surfaces and thus limit the development of mature biofilms [38].

### 3.5. Haemolytic Activity

**Figure 4:**
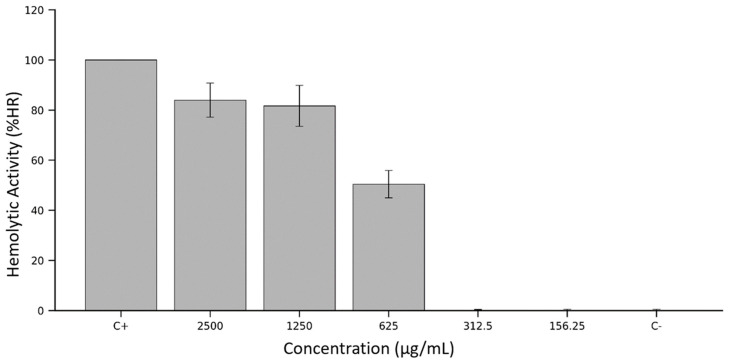
Haemolytic activity of *Calathea inocephala* (shutupipanga) freeze-dried extract against sheep erythrocytes. Erythrocytes were incubated for one hour at 37 °C with serial concentrations of the leaf extract (2500–156.25 µg mL^−1^). Haemoglobin released into the supernatant was quantified spectrophotometrically and expressed as percent haemolysis (%HR) after normalisation to the Triton X-100 positive control (C+) and the PBS negative control (C−). Bars represent the mean ± SD of three independent experiments (each performed in triplicate).

The *Calathea inocephala* (shutupipanga) freeze-dried extract produced a steep, concentration-dependent rise in erythrocyte lysis (Figure 4). Weighted optical-density values (OD_pond_) converted to percent haemolysis (%HR) showed that the extract was virtually equipotent with 10% Triton X-100 at the two highest doses, such as 83.97 ± 6.80% at 2500 µg mL^−1^ and 81.65 ± 8.19% at 1250 µg mL^−1^. Reducing the concentration to 625 µg mL^−1^ halved the response (50.42 ± 5.46%), while further dilutions (312.5 and 156.25 µg mL^−1^) were indistinguishable from the PBS control (≤0.5% HR). The narrow error bars at sublytic doses underscore the assay’s reproducibility and identify ~600 µg mL^−1^ as a critical threshold below which the extract is haemocompatible.

Targeted compositional analysis places caffeic acid (~17 g 100 g^−1^ DW) at the apex of the extract’s phenolic profile, followed by quercetin and kaempferol aglycones (≈0.7 g 100 g^−1^ DW in total). Caffeic acid can intercalate into lipid bilayers and, under oxidative conditions, initiate radical chain reactions that compromise membrane integrity [39]. At ≥1 mg mL^−1^, it induces >80% haemolysis of human erythrocytes in vitro.

Quercetin displays a dual behaviour: protective at sub-micromolar levels but haemolytic at >50 µM through pro-oxidant redox cycling in the presence of transition metals [40]. The maintenance of ~80% haemolysis on a two-fold dilution of shutupipanga extract, therefore, fits well with the concentration range at which these phenolics switch from membrane-stabilising to membrane-disruptive agents [41].

Pigments appear to reinforce this effect. The extract contains ~0.28 g 100 g^−1^ DW of chlorophyll-derived pheophytins and ~0.12 g 100 g^−1^ DW of lutein. Chlorophylls, pheophytins, and other tetrapyrroles are efficient photosensitisers that generate singlet oxygen and trigger photohaemolysis of erythrocytes under illumination; pheophytin formulations themselves can cause measurable haemolysis in vitro [42]. In our assay, incubation occurred in ambient laboratory light, providing sufficient photon flux to induce pigment-mediated oxidative stress and accounting for the near-Triton lysis at ≥1.25 mg mL^−1^.

Phenolic-rich botanicals often show a biphasic erythrocyte response. Green tea catechin mixtures provoke >80% haemolysis above 1 mg mL^−1^ but are protective below 0.2 mg mL^−1^. A *Thymus vulgaris* extract, containing comparable levels of chlorogenic and caffeic acids, produces ~50% haemolysis at 500 µg mL^−1,^ yet none at 100 µg mL^−1^. Conversely, *Lepidium aucheri* phenolics inhibit AAPH-induced haemolysis by ≈48% at 250 µg mL^−1^ but lose that protection as the dose rises. These parallels reinforce the view that shutupipanga’s abrupt toxicity threshold reflects a common phenolic-pigment synergy rather than an idiosyncratic metabolite.

Below ~300 µg mL^−1^, the extract is essentially haemocompatible; above ~600 µg mL^−1^, it becomes vigorously lytic. Any envisaged nutraceutical or pharmacological use must therefore respect this narrow safety window or involve fractionation to remove the pro-oxidant subset of phenolics and pigments. Future work should (i) isolate the caffeic-acid/pheophytin complex, (ii) test haemolysis under strictly dark conditions to parse phototoxic from intrinsic effects, and (iii) extend cytotoxic profiling to nucleated human cells to determine whether the erythrocyte threshold predicts broader cellular tolerance.

## 4. Conclusions

The leaves of bijao (*Calathea lutea*) and shutupipanga (*Calathea inocephala*) have traditionally been used by Amazonian communities for culinary purposes, mainly to wrap foods such as meat during cooking. In this study, it was determined that *C. lutea* had a slightly neutral pH, with high total titratable acidity and ash content, and that iron was among the most abundant minerals. It also showed a considerable concentration of vitamin C and organic acids, with tartaric acid predominating. Phytochemical screening revealed that *C. inocephala* tested positive for steroids, terpenoids, phenols, tannins, flavonoids, and acetogenins. Quantitative analysis showed high concentrations of carotenoids, especially lutein and β-carotene, as well as elevated levels of chlorophyll b and pheophytin b. In terms of phenolic compounds, high concentrations of gallic acid, caffeic acid, kaempferol, quercetin, and quercetin glycoside were identified, which are compounds widely associated with antioxidant and antimicrobial properties. The extracts evaluated demonstrated antimicrobial activity against all ATCC microorganisms tested, except Pseudomonas aeruginosa. Noteworthy was the inhibition of multidrug-resistant bacteria, such as *Enterococcus faecalis* and *Staphylococcus epidermidis*, with low minimum inhibitory concentrations (MIC). In terms of antibiofilm activity, *C. inocephala* showed effective inhibition against *Listeria monocytogenes* at low concentrations. Antioxidant activity, evaluated using the ABTS and DPPH methods, showed a slight superiority of *C. inocephala* over *C. lutea*. Meanwhile, haemolytic activity indicated a critical threshold near 600 µg/mL, below which the extract can be considered haemocompatible. These results constitute a relevant starting point that highlights the importance of further studies on these species, incorporating more specific tests that enable the sustainable use of *Calathea* leaves within Amazonian communities, thereby contributing to the rational use of biological resources and local economic strengthening.

## Figures and Tables

**Table 1:** Physicochemical properties of *Calathea lutea* and *Calathea inocephala* leaves.

Parameters	*Calathea lutea* (Bijao)	*Calathea inocephala* (Shutupipanga)
pH	6.8 ± 0.1 ^a^	5.8 ± 0.0 ^b^
Soluble solids (°Brix)	1.0 ± 0.0 ^a^	1.0 ± 0.0 ^a^
Total titratable acidity (%)	0.5 ± 0.0 ^a^	0.2 ± 0.1 ^a^
Humidity (%)	45.1 ± 6.6 ^b^	67.4 ± 0.3 ^a^
Ash (%)	4.6 ± 0.9 ^a^	2.3 ± 0.1 ^b^
Mineral profile (mg/100 g DW)		
Ca	103.2 ± 10.7 ^b^	176.9 ± 14.3 ^a^
Fe	2930.0 ± 85.4 ^a^	1955.4 ± 62.3 ^b^
K	197.4 ± 22.6 ^a^	158.0 ± 6.1 ^b^
Mg	15.1 ± 2.3 ^a^	6.6 ± 0.5 ^b^
Na	47.1 ± 0.1 ^b^	55.4 ± 0.4 ^a^

Note: Different lowercase letters indicate significant differences between the two species in the study.

**Table 2:** Qualitative determination of secondary metabolites of *Calathea lutea* and *Calathea inocphela* leaves.

Metabolite Secondary	*Calathea lutea* (Bijao)	*Calathea inocephala* (Shutupipanga)
Steroids	−	+
Terpenoids	−	+
Phenols	+	+
Tannins	+	+
Alkaloids	−	−
Flavonoids	−	+
Anthraquinones	−	−
Saponins	−	−
Acetoginins	+	+

Note: −, negative test result; +, positive test result.

**Table 3:** Average concentration of bioactive compounds of *Calathea lutea* and *Calathea inocephala* leaves.

Parameters	*Calathea lutea* (Bijao)	*Calathea inocephala* (Shutupipanga)
Vitamin C (mg/100 g DW)	4.6 ± 0.0 ^a^	2.7 ± 0.4 ^b^
Organic acid profile (mg/100 g DW)		
Citric acid	244.9 ± 10.7 ^a^	66.7 ± 16.1 ^b^
Malic acid	22.3 ± 0.1 ^a^	16.4 ± 2.3 ^b^
Tartaric acid	722.3 ± 48.8 ^a^	4.0 ± 0.3 ^b^
Total organic acid	989.6 ± 59.4 ^a^	87.0 ± 13.6 ^b^
Carotenoid profile (mg/100 g DW)		
Lutein	7.4 ± 1.4 ^b^	83.5 ± 2.0 ^a^
Zeaxanthin	0.9 ± 0.1 ^b^	2.5 ± 0.1 ^a^
Zeionaxanthin	0.5 ± 0.0 ^b^	0.8 ± 0.1 ^a^
α-carotene	nd	2.2 ± 0.0
β-carotene	nd	26.2 ± 1.4
Total carotenoid	8.8 ± 1.5 ^b^	115.1 ± 0.5 ^a^
Chlorophylls and their derivatives (mg/100 g DW)		
Chlorophyll b	54.2 ± 5.7 ^b^	101.4 ± 1.3 ^a^
Pheophytin a	5.9 ± 0.8	nd
Pheophytin b	9.1 ± 0.6 ^b^	177.5 ± 0.2 ^a^
Total chlorophylls	69.2 ± 1.2 ^b^	278.9 ± 1.1 ^b^
Phenolics profile (mg/100 g DW)		
Gallic acid	10.9 ± 0.2 ^b^	407.7 ± 4.6 ^a^
4-Hydroxybenzoic acid	59.9 ± 1.6	nd
Syringic acid	105.3 ± 6.6	nd
Chlorogenic acid	371.6 ± 41.7 ^a^	201.4 ± 2.9 ^b^
Caffeic acid	586.2 ± 57.7 ^b^	16,996.3 ± 24.7 ^a^
Ferulic acid	500.1 ± 24.2	nd
Rutin	29.4 ± 0.7	nd
Kaempferol	76.5 ± 2.5 ^b^	667.2 ± 12.2 ^a^
Quercetin glycoside	25.2 ± 0.1 ^b^	335.7 ± 5.7 ^a^
Quercetin	20.5 ± 0.6 ^b^	390.1 ± 5.7 ^a^
Total phenolics	1785.6 ± 135.7 ^b^	18,998.4 ± 278.2 ^a^
Antioxidant activity (mmol TE (100 g DW)		
ABTS	4.1 ± 0.9 ^a^	4.4 ± 0.9 ^a^
DPPH	3.9 ± 0.0 ^b^	4.6 ± 0.0 ^a^

Note: nd, undetectable. Different lowercase letters indicate significant differences between the two species in the study.

**Table 4:** Antimicrobial activity of *Calathea lutea* and *Calathea inocephala* freeze-dried extract.

Extracts	Zone of Inhibition (mm)					
	*Bacterial strain*				*Fungal strain*	
	*E. coli* ATCC 8739	*S. aureus* ATCC 6538P	*P. aeruginosa* ATCC 9027	*S. mutans* ATCC 25175	*C. albicans* ATCC 1031	*C. tropicalis* ATCC 13803
*C. lutea* (Bijao)	16.5 ± 2.1	16.5 ± 0.1	-	12.5 ± 0.71	8.8 ± 0.1	11.0 ± 0.2
*C. inocephala* (Shutupipanga)	19.0 ± 0.0	23.0 ± 0.0	10.0 ± 1.4	32.0 ± 1.41	10.1 ± 0.0	14.5 ± 0.1
Control *	26.2 ± 1.55	22.5 ± 3.27	25.0 ± 1.7	31.1 ± 1.49	10.6 ± 2.37	16.8 ± 2.20

Note: -: non-active at the tested concentration; * streptomycin for bacteria and fluconazole for fungi.

**Table 5:** Minimal inhibitory concentration of *Calathea lutea* and *Calathea inocephala* extract.

Microorganisms	Minimal Inhibitory Concentration (mg/mL)	
	*C. lutea*	*C. inocephala*
*E. coli* ATCC 8739	10.73	21.04
*P. aeruginosa* ATCC 9027	-	85.83
*S. aureus* ATCC 6538P	21.46	10.52
*S. mutans* ATCC 25175	21.46	21.04
*C. albicans* ATCC	85.83	10.52
*C. tropicalis* ATCC	85.83	10.52

Note: -: non-active at the tested concentration.

**Table 6:** Minimal inhibitory concentration of *Calathea inocephala* freeze-dried extract.

Bacteria Strain	Minimal Inhibitory Concentration (mg/mL)
*Enterococcus faecalis*	1.00
*Enterococcus faecium*	4.00
*Escherichia coli*	-
*Klebsiella pneumoniae*	-
*Pseudomonas aeruginosa*	-
*Staphylococcus epidermidis*	1.00
*Salmonella enterica serovar Kentucky*	-

## Data Availability

The original contributions presented in this study are included in the article. Further inquiries can be directed to the corresponding author.
